# Immune correlates of aging in outdoor-housed captive rhesus macaques (*Macaca mulatta*)

**DOI:** 10.1186/1742-4933-9-25

**Published:** 2012-11-14

**Authors:** Elizabeth S Didier, Chie Sugimoto, Lisa C Bowers, Imtiaz A Khan, Marcelo J Kuroda

**Affiliations:** 1 Division of Microbiology, Tulane National Primate Research Center, 18703 Three Rivers Road, Covington, LA, 70433, USA; 2 Department of Tropical Medicine, School of Public Health and Tropical Medicine, Tulane University, New Orleans, New Orleans, LA, 70112, USA; 3 Division of Immunology, Tulane National Primate Research Center, 18703 Three Rivers Road, Covington, LA, 70433, USA; 4 Department of Microbiology, Immunology, and Tropical Medicine, Ross Hall Room 745, George Washington University, 2300 I Street, N.W, Washington D.C, 20037, USA

**Keywords:** Inflammation, Inflamm-aging, Cytokine, Chemokine, Multiplex, Rhesus macaque, Aging, Animal model, Immune senescence, Blood

## Abstract

**Background:**

Questions remain about whether inflammation is a cause, consequence, or coincidence of aging. The purpose of this study was to define baseline immunological characteristics from blood to develop a model in rhesus macaques that could be used to address the relationship between inflammation and aging. Hematology, flow cytometry, clinical chemistry, and multiplex cytokine/chemokine analyses were performed on a group of 101 outdoor-housed captive rhesus macaques ranging from 2 to 24 years of age, approximately equivalent to 8 to 77 years of age in humans.

**Results:**

These results extend earlier reports correlating changes in lymphocyte subpopulations and cytokines/chemokines with increasing age. There were significant declines in numbers of white blood cells (WBC) overall, as well as lymphocytes, monocytes, and polymorphonuclear cells with increasing age. Among lymphocytes, there were no significant declines in NK cells and T cells, whereas B cell numbers exhibited significant declines with age. Within the T cell populations, there were significant declines in numbers of CD4+ naïve T cells and CD8+ naïve T cells. Conversely, numbers of CD4+CD8+ effector memory and CD8+effector memory T cells increased with age. New multiplex analyses revealed that concentrations of a panel of ten circulating cytokines/chemokines, IFNγ, IL1b, IL6, IL12, IL15, TNFα, MCP1, MIP1α, IL1ra, and IL4, each significantly correlated with age and also exhibited concordant pairwise correlations with every other factor within this group. To also control for outlier values, mean rank values of each of these cytokine concentrations in relation to age of each animal and these also correlated with age.

**Conclusions:**

A panel of ten cytokines/chemokines were identified that correlated with aging and also with each other. This will permit selection of animals exhibiting relatively higher and lower inflammation status as a model to test mechanisms of inflammation status in aging with susceptibility to infections and vaccine efficacy.

## Background

The elderly are at higher risk of suffering from morbidity and mortality associated with infectious diseases and cancer, especially as immune competence wanes. Immune senescence also contributes to a decline in vaccine efficacy in the elderly that poses a tremendous challenge to public health, particularly as the population of persons over 65 years of age is growing [1-3]. Defining immunologic markers that predict an individual’s resistance or susceptibility to age-related illness is expected to improve implementation of public and medical health measures. A hallmark of immune senescence is chronic inflammation or “inflamm-aging”, but it is unclear if or how chronic inflammation in the elderly contributes to increasing susceptibility to infections and cancer, or declines in vaccine efficacy [4-6].

Nonhuman primate rhesus macaques (*Macaca mulatta*) are commonly used in biomedical research because of they are phylogenetically and physiologically related to humans [7]. Macaques are susceptible to nearly identical infections and diseases as humans, and thus are important for preclinical vaccine and drug testing prior to application in humans. The average and maximum lifespans of rhesus macaques are approximately 25 years and 40 years, respectively [8]. Humans in resource-rich countries average approximately 80 years in life-span with a maximal survival time reported at 120 years, that represents an estimated 3.2-fold difference for relating age between humans and rhesus macaques [5,8,9].

In this report, a cross-sectional experimental design was applied to characterize basic clinical and immunological correlates in blood specimens of outdoor-housed rhesus macaques ranging from 2.2 to 24 years of age at the Tulane National Primate Research Center in Covington, LA USA. Levels of blood cell populations and circulating cytokines/chemokines were identified that significantly correlated individually and in combinations with age. The results provide a model to compare chronologically older nonhuman primates exhibiting relatively higher levels of “inflamm-aging” cytokines/chemokines with age-matched animals expressing relatively lower levels of these cytokines/chemokines to test hypotheses relating chronic inflammation to vaccine efficacy as well as susceptibility from infectious diseases and cancer during aging.

## Methods

### Nonhuman primates, venipuncture, hematology, and blood chemistry

Rhesus macaques (*Macaca mulatta*) were housed in outdoor field cages at the Tulane National Primate Research Center in Covington, LA USA. Procedures for venipuncture and physical examination were performed during biannual preventive medicine evaluations under anesthesia to minimize stress and were approved by the Institutional Animal Care and Use Committee of Tulane University in accordance with the Guide for the Care and Use of Laboratory Animals of the National Institutes of Health (NIH). The rhesus macaques used in this study are part of the specific pathogen-free colony (SPF) and as part of the biannual preventive medicine surveillance, are tested twice per year to assure that they are seronegative for SIV, SRV, Herpes B virus and STLV and PCR negative for SRV. Routine clinical examinations (bacteriology, parasitology, hematology, blood chemistry) are performed as well, and animals are treated accordingly. Hematology was performed on EDTA-preserved blood using the Advia 120 instrument (Siemens Healthcare Diagnostics, Inc., Tarrytown, NY) and serum clinical chemistry was performed using the Beckman Olympus AU400e (Brea, CA).

### Flow cytometry

EDTA-preserved whole blood was stained for flow cytometric analysis, and the following mAbs were used in this study: CD3 (Alexa Fluor 700 and Pacific Blue, clone SP34-2, BD Biosciences, San Jose, CA), CD4 (PerCP-Cy5.5, clone L200, BD Biosciences), CD8 (AmCyan, clone SK1, BD Biosciences), CD20 (APC-H7, clone 2H7, BD Biosciences), CD20 (ECD, clone B9E9, Beckman Coulter, Brea, CA), CD28 (ECD, clone 28.1, Beckman Coulter), CD95 (APC, clone DX2, BD Biosciences), TCR Vδ2 (FITC, clone 15D, Pierce Biotechnology, Rockford, IL), TCR-γδ (PE, clone SA6.E9, Invitrogen, Life Technology, Grand Island, NY) and NKG2a (APC, clone Z199, Beckman Coulter). One hundred microliters of blood were stained with antibodies for 20 min at the room temperature and red blood cells (RBC) were then lysed with 1× FACS lysing solution (BD Biosciences). After washing the cells twice with phosphate-buffered saline (PBS) containing 2% FBS, the cells were fixed with PBS containing 1% formaldehyde (Sigma, St. Louis, MO). Results were acquired on a LSR II (BD Biosciences) and analyzed using FlowJo software (TreeStar, Inc., Ashland, OR).

### Cytokine quantification

EDTA-preserved plasma samples were centrifuged (14,000 × g for 5 minutes) and aliquots were frozen at −80°C until used. Prior to assay, once-thawed plasma samples were pre-cleared using Ultrafree Centrifugal Filters (Millipore, Billerica, MA). Cytokine levels were measured using the Milliplex MAP Non-Human Primate Cytokine Panel (Milllipore, Billerica, MA) or the Monkey Cytokine Magnetic 28-Plex Panel (Invitrogen, Life Technologies) according to manufacturer’s instructions. The reactions in microtiter plates were read on a Bioplex-200 system instrument and results were calculated using BioPlex software version 6 (BioRad, Hercules, CA).

### Statistical analyses

Pearson correlation coefficients were calculated to compare each test variable in relation to age of the monkeys. Paired comparisons between means of specified groups were calculated by Student’s *t* Test, and Fisher’s r-to-z transformation was performed to compare correlation coefficients between males and females. Analyses were performed and graphed using Graphpad Prism version 5.00 for Windows (GraphPad Software, San Diego California USA, http://www.graphpad.com), and *P* < 0.05 was considered significant.

## Results

### Study group

The study population consisted of 101 rhesus macaques ranging from 2 – 24 years of age (Table 1) and included 22 males and 79 females comparably distributed between three groups ranging from 2 – 9 (younger), 10 – 17 (middle), and 18 – 24 (older) years of age. Based on an estimated 3.2-fold age differential between rhesus macaques and humans [5,8,9], the age ranges of the study groups of monkeys were approximately equivalent to humans of 8 –30 years in the younger group, 31 – 56 years in the middle group, and 57 – 77 years in the older group. There was no significant difference in the mean ages of males and females used in the study. Animals greater than 24 years of age were not included in this report to preclude effects of chronic diseases of aging on immune correlates in blood and to prevent skewing of results from death of frail older animals.

**Table 1:** Study group of rhesus macaques

**Study group**	**Males**	**Females**	**Total**
**Age of monkeys**			
**(human age equivalent)**^**a**^			
**Younger NHPs**	7	25	32
2 – 9 years			
(7 – 29 years)			
**Middle-age NHPs**	7	28	35
10 – 17 years			
(32 – 55 years)			
**Older NHPs**	8	26	34
18 – 24 years			
(58 – 77 years)			
**Total**	22	79	101
**Mean age**	13.41	13.15	13.21
**St. dev**.	7.08	5.41	5.77

^a^ Equivalent human ages were estimated as a 3.2-fold difference from rhesus macaque ages.

### Hematology

Correlation coefficients between hematology values and age overall, as well as comparisons between mean values of the three age groups are shown in Table 2. Among the erythrocyte-related tests [red blood cells (RBC), hemoglobin (Hgb), hematocrit (Hct), mean corpuscular volume (MCV), mean corpuscular hemoglobin (MCH), mean corpuscular hemoglobin concentration (MCHC), and red blood cell distribution (RDW)], there were no statistically significant correlation coefficients with age and no statistically significant differences between the younger and older age groups. Mean Hct levels (12.23 ± 1.26 vs 13.05 ± 1.01 g/dL; *P* = 0.006) and MCV values (37.96 ± 5.50 vs 40.53 ± 2.83 %; *P* = 0.0374) were significantly lower in females than males, respectively, but there were no significant differences in correlation coefficients over age between males and females using r-to-z transformation. Numbers of platelets and MPV did not exhibit statistically significant correlations with age, and there were no significant differences in mean values between males and females within each age group or after comparing correlation coefficients between genders.

**Table 2:** Relationship between hematology values and age in rhesus macaques

**Test**^**a**^	**Correlation coefficient (r)**^**b**^	***P***	**Mean values (± St. Dev.)**				
			**Younger group (2–9 years)**	**Middle group 10**–**17 years**	**Older group 18**–**24 years**	***P***^**c**^	**Units**
RBC	−0.1359	n.s.^d^	5.64 ± 0.44	5.59 ± 0.53	5.5 ± 0.61	n.s.	x 10^6^ / μL
Hgb	−0.1145	n.s.	12.58 ± 0.85	12.59 ± 1.31	12.3 ± 1.3	n.s.	g / dL
Hct	−0.1276	n.s.	39.47 ± 3.22	39.22 ± 3.55	37.96 ± 6.48	n.s.	%
MCV	0.0766	n.s.	70.01 ± 2.85	70.35 ± 3.55	70.49 ± 3.79	n.s.	fL
MCH	−0.0011	n.s.	22.33 ± 0.96	22.60 ± 1.61	22.28 ± 1.79	n.s.	pg/cell
MCHC	−0.1029	n.s.	31.89 ± 0.94	32.11 ± 1.17	31.58 ± 1.59	n.s.	g / dL
RDW	0.0055	n.s.	13.49 ± 1.00	13.22 ± 2.62	13.61 ± 1.25	n.s.	%
Platelets	−0.1364	n.s.	4.04 ± 0.89	3.86 ± 0.93	3.69 ± 1.20	n.s.	x 10^5^ / μL
MPV	0.0613	n.s.	9.28 ± 0.92	8.90 ± 2.17	9.19 ± 1.311	n.s.	fL
WBC	−0.1768	0.0380	10.68 ± 5.97	9.72 ± 4.56	6.68 ± 2.94	0.0007	x 10^3^ / μL
Lymphocytes	−0.3052	0.0002	2.78 ± 1.18	2.70 ± 1.30	2.12 ± 0.66	0.0052	x 10^3^ / μL
Monocyte	−0.1989	0.0170	0.47 ± 0.24	0.42 ± 0.16	0.36 ± 0.14	0.0221	x 10^3^ / μL
Neutrophils	−0.2555	0.0020	7.07 ± 5.72	6.01 ± 4.38	4.03 ± 2.96	0.0068	x 10^3^ / μL
Eosinophils	−0.2710	0.0010	0.36 ± 0.29	0.29 ± 0.26	0.18 ± 0.15	0.0017	x 10^3^ / μL
Basophils	−0.3525	< 0.0001	0.04 ± 0.03	0.03 ± 0.03	0.02 ± 0.01	0.0004	x 10^3^ / μL

^a^ Abbreviations of tests; RBC, red blood cells; Hgb, hemoglobin, Hct, hematocrit; MCV, mean corpuscular volume; MCH, mean corpuscular hemoglobin; MCHC, mean corpuscular hemoglobin concentration; RDW, red blood cell distribution width; MPV, mean platelet volume; WBC, white blood cells.

^b^ Pearson correlation coefficients were calculated for each hematology test over age of the rhesus macaques. *P* < 0.05 was considered statistically significant.

^c^ Mean values of hematology tests were calculated for all animals and within groups of rhesus macaques. Student’s *t* Test was measured to determine statistically significant differences between the older and younger groups of animals. *P* < 0.05 was considered statistically significant.

^d^ Not significant, n.s.

Significant inverse correlations were observed between numbers of white blood cells (WBC), polymorphonuclear cells (neutrophils, basophils, and eosinophils) and mononuclear cells (monocytes and lymphocytes) with age. In addition, mean levels of these blood cell populations were statistically significantly lower in the older group compared with respective means in the younger group of animals. Comparisons of mean values within each age group revealed no statistically significant differences between males and females. Correlation coefficients were compared between genders using r-to-z transformation and were significantly lower in females than males for declining levels of circulating WBC (Z = −2.85; p = 0.0044) and neutrophils (Z = −2.02; *P* = 0.0434) with increasing age.

### Lymphocyte subpopulations in blood

**Figure 1:**
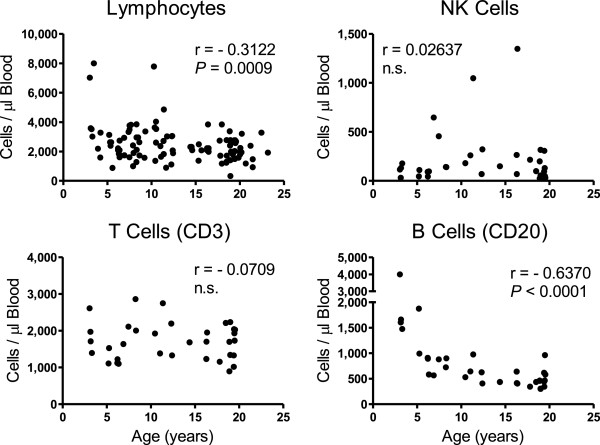
**Relationships between peripheral blood mononuclear cells and age of rhesus macaques.** Peripheral blood lymphocyte values were obtained as part of the CBC measurements from all animals in the study group. Flow cytometry was applied to measure NK cells (CD3-CD20-CD8+NKG2a+), T cells (CD3+CD20-) and B cells (CD3-CD20+) from a subset of 34 monkeys of the study group. Pearson correlation coefficients (r values) were calculated between cell number and age of the animals, and *P* < 0.05 was considered statistically significant.

A statistically significant inverse correlation (r = −0.3122; *P* = 0.0009) was observed between circulating numbers of lymphocytes with age of rhesus macaques, and flow cytometry was then applied to evaluate lymphocyte subset populations from 34 of the rhesus macaques (Figure 1). No statistically significant correlations were seen in levels of circulating CD3+ T cells or NK cells in relation to age, but a statistically significant inverse correlation was observed between CD20+ B cells with increasing age (r = −0.6370; *P* < 0.0001). There were no statistically significant differences in correlation coefficients or mean levels of the lymphocyte populations between males and females.

**Figure 2:**
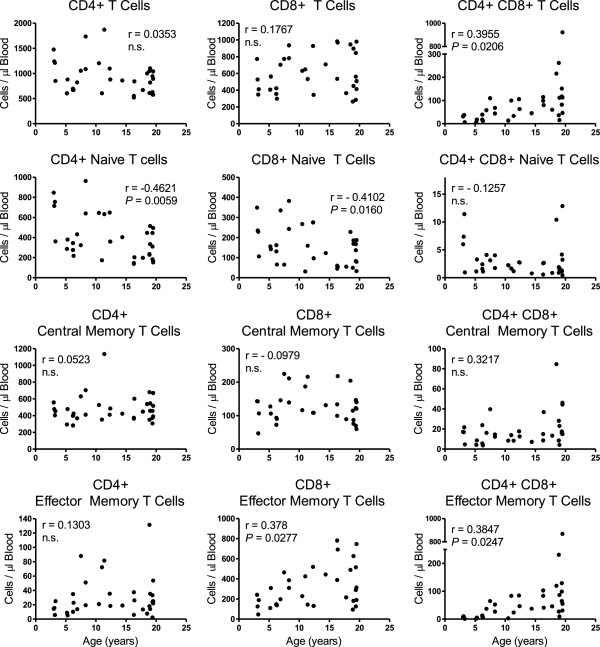
**Relationships between T cell subpopulations and age of rhesus macaques.** Blood was recovered from 34 rhesus macaques, stained, and analyzed for T cell subpopulations by flow cytometry. Naïve cells were CD28+ and CD95-, central memory cells were CD28+ and CD95+, and effector memory cells were CD28- and CD95+ [10]. Pearson correlation coefficients were calculated between cell number and age of the animals, and *P* < 0.05 was considered statistically significant.

T cell subpopulation levels that were further evaluated by flow cytometry are shown in Figure 2. As shown in the left panel of graphs, no statistically significant correlation was observed between CD4+ T cells overall and age. Within the CD4+ T cell population, however, a significant inverse correlation was measured between CD4+ naïve T cells and age (r = −0.4261; *P* = 0.0059) whereas no statistically significant correlations were observed between CD4+ effector memory or CD4+ central memory T cells and age. There were no statistically significant gender differences in mean levels or correlation coefficients of CD4+ T cells overall or subset populations.

No statistically significant correlations were measured between CD8+ T cells overall or CD8+ central memory T cells with age as shown in the middle panel of Figure 2. A statistically significant inverse correlation was observed between CD8+ naïve T cells over age (r = −0.4102; *P* = 0.0160), and a statistically significant direct correlation was seen between CD8+ effector memory T cells and age (r = 0.378; *P* = 0.0277) that also was greater among males than females (Z = 3.13; *P* = 0.0017). There were no statistically significant differences, however, in mean levels of the CD8+ T cell subpopulations between genders.

Statistically significant direct correlations were observed between CD4+CD8+ double-positive (DP) T cells overall (r = 0.3955; P = 0.0206) and DP effector memory T cells (r = 0.3847; P = 0.0247) over age (Figure 2, right panel). No statistically significant correlations were measured between DP central memory and DP naïve cells with age. There also were no statistically significant differences between genders when comparing means of each of the DP T cell populations. Males, however, exhibited significantly higher correlation coefficients than females for DP T cells overall (*Z* = 1.99; *P* 0.0477) and DP effector memory T cells (*Z* = 2.21; *P* = 0.0271) over age. Levels of TCR δ2+ γδ T cells and TCR δ2- γδ T cells exhibited no statistically significant correlation with age and there were no significant differences between genders when comparing mean levels of these cells or correlation coefficients (not shown).

### Blood chemistry

Blood chemistry analyses of this study population are presented in Table 3 and statistically significant inverse correlations were observed for albumin (r = −0.2276; *P* = 0.0410), creatinine (r = −0.2763; *P* = 0.0310), and aspartate transaminase (r = −0.3126; *P* = 0.005) with increasing age. Mean aspartate transaminase levels were significantly lower in the older group of 18–24 year-old animals compared to the younger 2–9 year-old group. Males expressed significantly lower correlation coefficients than females for albumin (*Z* = −2.30; *P* = 0.0214) and albumin/globulin (*Z* = −3.17; *P* =0.0015) with age. Conversely, females expressed a significantly lower correlation coefficient than males for creatinine (*Z* = −2.60; *P* = 0.0093) with age. No significantly different correlation coefficients were observed between genders for the other blood chemistry values measured.

**Table 3:** Relationship between blood chemistry values over age in rhesus macaques

**Test**	**Correlation coefficient (r)**^**a**^	***P***	**Mean values (± St. Dev.)**				
			**Younger group (2–9 years)**	**Middle group (10–17 years)**	**Older group (18–24 years)**	***P***^**b**^	**Units**
Sodium (Na)	−0.0835				n.s.^c^	147.80 ± 3.23	147.04 ± 3.55	147.40 ± 3.41	n.s.	mMol/L
Potassium (K)	−0.0278				n.s.	3.86 ± 0.60	3.65 ± 0.36	3.90 ± 0.45	n.s.	mMol/L
Chloride (Cl)	0.1830				n.s.	109.00 ± 2.98	108.42 ± 5.16	110.70 ± 4.03	n.s.	mMol/L
Total protein	−0.2026				n.s.	7.01 ± 0.60	6.95 ± 0.56	6.80 ± 0.75	n.s.	g/dL
Albumin	−0.2276				0.041	3.83 ± 0.05	3.75 ± 0.50	3.60 ± 0.69	n.s.	g/dL
Globulins	0.1124				n.s.	3.18 ± 0.58	3.20 ± 0.52	3.10 ± 0.55	n.s.	g/dL
Albumin/Globulins	−0.1284				n.s.	1.26 ± 0.36	1.22 ± 0.28	1.20 ± 0.35	n.s.	
Blood Urea Nitrogen (BUN)	−0.2017				n.s.	22.05 ± 9.47	21.17 ± 5.98	19.30 ± 7.33	n.s.	mg/dL
Creatinine	−0.2763				0.031	0.88 ± 0.19	0.89 ± 0.28	0.80 ± 0.32	n.s.	mg/dL
BUN/Creatinine	0.1373				n.s.	25.52 ± 8.94	24.93 ± 7.09	27.60 ± 8.89	n.s.	
Glucose	0.1240				n.s.	60.72 ± 19.22	68.23 ± 22.84	64.40 ± 23.59	n.s.	mMol/L
Alanine Transaminase	−0.0125				n.s.	29.04 ± 14.66	32.35 ± 14.52	36.90 ± 18.34	n.s.	IU/L
Aspartate Transaminase	−0.3126				0.005	46.63 ± 18.38	37.88 ± 16.03	37.50 ± 18.38	0.0415	IU/l

^a^ Pearson correlation coefficients were calculated for each hematology test over age of the rhesus macaques. *P* < 0.05 was considered statistically significant.

^b^ Mean values of hematology tests were calculated for animals in the three age groups of rhesus macaques. Student’s *t* Test was measured to determine statistically significant differences between the older and younger groups of animals. *P* < 0.05 was considered statistically significant.

^c^ Not significant = n.s.

### Plasma cytokines

**Figure 3:**
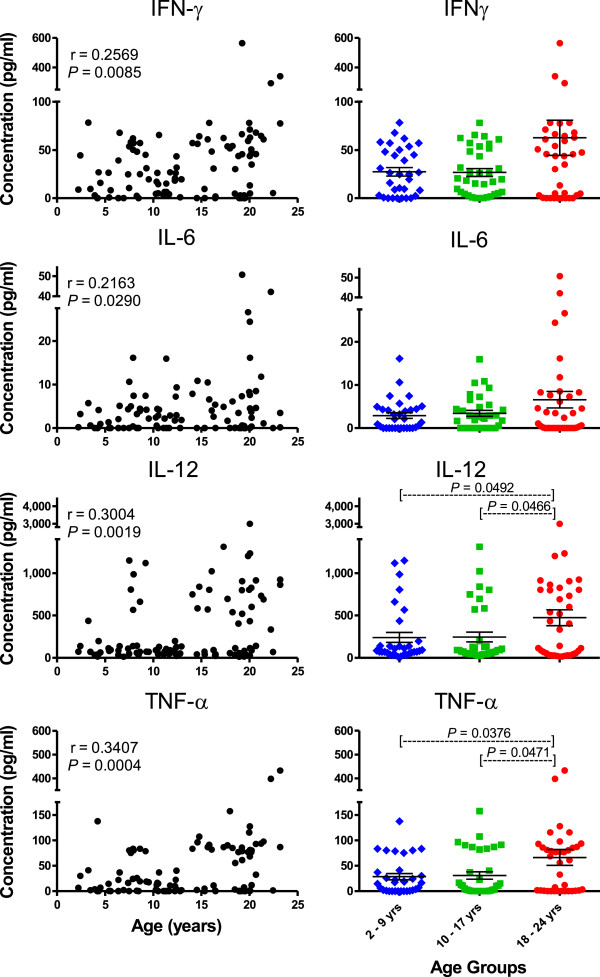
**Relationships between concentrations of selected circulating pro-inflammatory cytokines and age of each rhesus macaque in the study group.** Pearson correlation coefficients were calculated between each circulating cytokine concentration and age as shown in the left panel. Student’s *t* Test was used to compare means of circulating cytokine concentrations between the three age groups of monkeys. *P* < 0.05 was considered statistically significant.

Correlation coefficients and mean plasma concentrations of cytokines in the younger, middle, and older age groups of rhesus macaques are shown in Table 4. Among the pro-inflammatory cytokines, IFNγ, IL1β, IL6, IL12/IL23(p40), IL15, MIF, sCD40L, and TNFα, exhibited statistically significant direct correlations with age whereas circulating levels of IL17 did not correlate with age. In addition, mean levels of circulating IL1β, IL12/IL23(p40), sCD40L, and TNFα were statistically significantly higher in the older group of monkeys aged 18 – 24 years of age than in the younger group of monkeys aged 2 – 9 years of age. Results of four pro-inflammatory cytokines commonly associated with aging, IFNγ, IL6, IL12/IL23(p40), and TNFα were plotted in Figure 3. Of interest was the greater variability of cytokine levels in the older group of animals. For example, circulating levels of IFNγ and IL6 exhibited statistically significant direct correlations with age, but mean levels in the younger and older groups were not statistically significantly different due to the high standard deviations in the older group. Among all the pro-inflammatory cytokines assayed and listed in Table 4, there were no significant differences when comparing mean concentrations between the younger age group and middle age groups, suggesting that the shifts to higher expression of these cytokines occurred during the transition from the middle to older age groups. Mean concentrations and correlation coefficients of these pro-inflammatory cytokines over age did not significantly differ between genders.

**Table 4:** Relationship between plasma cytokine levels and age in rhesus macaques

**Category cytokine**	**All animals**		**Mean concentration ± st. dev. (pg/ml)**			***P***^**b**^
	**Correlation coefficient ( r )**^**a**^	***P***	**Younger group (2–9 years)**	**Middle group (10–17 years)**	**Older group (18–24 years)**	
**Pro-inflammatory / Th1 induction:**						
**IFN-γ**	0.2569	0.0085	27.29 ± 24.40	26.59 ± 24.09	59.99 ± 107.04	n.s.^c^
**IL-1β**	0.2704	0.0055	10.06 ± 14.18	10.19 ± 15.57	16.67 ± 18.18	0.0457
**IL-6**	0.2163	0.0290	2.89 ± 3.69	3.42 ± 3.94	6.18 ± 11.31	n.s.
**IL-12/IL-23 (p40)**	0.3004	0.0019	240.91 ± 335.77	245.70 ± 339.13	443.50 ± 564.21	0.0492
**IL-15**	0.2792	0.0045	11.25 ± 9.05	11.55 ± 8.80	16.05 ± 13.57	n.s.
**IL-17**	0.0281	n.s.	2.81 ± 5.72	2.15 ± 5.20	2.97 ± 12.88	n.s.
**MIF**	0.5151	0.0036	130.19 ± 61.89	371.06 ± 293.29	624.15 ± 598.64	n.s.
**sCD40L**	0.3621	0.0016	2,731.76 ± 4,072.20	2,644.32 ± 3,646.08	5,874.23 ± 4,435.26	0.0025
**TNF-α**	0.3407	0.0004	28.40 ± 35.51	30.71 ± 42.78	62.28 ± 62.43	0.0376
**Chemokines:**						
**Eotaxin (CCL11)**	0.1865	n.s.	441.49 ± 92.46	738.88 ± 345.51	635.78 ± 370.24	n.s.
**IL-8 (CXCL8)**	0.2165	0.0280	1,273.54 ± 2,115.97	1,538.39 ± 2,287.07	3,970.65 ± 7,165.05	0.0345
**I-TAC (CXCL11)**	−0.0762	n.s.	111.86 ± 241.19	140.35 ± 198.89	92.71 ± 116.12	n.s.
**MCP-1 (CCL2)**	0.2644	0.0067	318.35 ± 176.44	338.75 ± 236.98	400.60 ± 190.78	0.0270
**MDC (CCL22)**	0.1279	n.s.	934.06 ± 626.64	1,657.17 ± 729.31	1,220.65 ± 1,113.48	n.s.
**MIG (CXCL9)**	−0.4799	0.0073	112.82 ± 24.23	120.31 ± 22.89	83.66 ± 13.81	0.0019
**MIP-1α (CCL3L1)**	0.2877	0.0031	23.64 ± 20.58	25.07 ± 22.63	36.69 ± 33.56	0.0466
**MIP-1β (CCL4)**	0.0786	0.0108	19.84 ± 19.56	15.33 ± 15.74	24.78 ± 37.63	n.s.
**RANTES (CCL5)**	0.2065	n.s.	16,617.28 ± 8,693.23	43,928.17 ± 61,521.00	38,343.38 ± 56,904.69	n.s.
**Growth factors:**						
**EGF**	0.6582	< 0.0001	33.80 ± 6.86	85.33 ± 126.15	321.85 ± 148.42	<0.0001
**FGF-basic**	0.4038	0.0269	13.24 ± 2.59	13.85 ± 2.14	20.05 ± 8.54	n.s.
**G-CSF**	0.3179	0.0011	73.69 ± 94.41	77.18 ± 94.45	115.94 ± 111.56	0.0258
**GM-CSF**	0.0162	n.s.	83.77 ± 247.97	93.94 ± 273.92	74.80 ± 210.17	n.s.
**HGF**	0.1149	n.s.	107.24 ± 23.63	148.00 ± 73.14	136.17 ± 107.81	n.s.
**IL-2**	0.4062	< 0.0001	75.93 ± 84.58	87.88 ± 72.09	162.49 ± 131.83	0.0004
**TGF-α**	0.2533	0.0318	9.28 ± 7.64	7.50 ± 6.30	13.75 ± 8.05	0.0258
**TGF-β1**	0.1896	n.s.	17,577.10 ± 7,978.62	16,257.61 ± 7,884.94	22,025.53 ± 8,156.91	n.s.
**TGF-β2**	0.1043	n.s.	2,252.57 ± 1,121.62	2,113.75 ± 1,141.93	2,493.74 ± 847.81	n.s.
**TGF-β3**	−0.3218	0.0457	27.66 ± 13.09	17.57 ± 9.15	19.46 ± 9.79	n.s.
**VEGF**	0.0380	n.s.	91.90 ± 164.42	77.34 ± 158.25	105.50 ± 169.99	n.s.
**Anti-inflammatory / B cell and Th2 induction:**						
**IL-1ra**	0.3533	0.0002	195.35 ± 393.17	212.41 ± 352.53	466.24 ± 559.77	0.0126
**IL-4**	0.3496	0.0003	73.18 ± 144.16	86.82 ± 157.78	170.45 ± 197.72	0.0120
**IL-5**	−0.3003	0.0022	4.45 ± 5.63	3.28 ± 4.46	1.51 ± 2.37	0.0021
**IL-10**	0.1017	n.s.	4.60 ± 4.33	5.73 ± 9.92	6.35 ± 9.52	n.s.
**IL-13**	0.2172	n.s.	2.37 ± 1.96	3.76 ± 4.62	4.05 ± 6.28	n.s.

^a^ Pearson correlation coefficients were calculated for each circulating cytokine concentration over age of the rhesus macaques. *P* < 0.05 was considered statistically significant.

^b^ Means of circulating cyotokine concentration were calculated for the three age groups of rhesus macaques. Student’s *t* Test was measured to determine statistically significant differences between the older and younger groups of animals. *P* < 0.05 was considered statistically significant.

^c^ n.s.; not significant.

Among the chemokines tested, circulating levels of IL8, MCP1, MIP1α, and MIP1β significantly correlated directly with age and with the exception of MIP-1β, mean concentrations of these factors were significantly higher in the older group than in the younger group of monkeys. Conversely, a significant inverse correlation was observed in levels of MIG with increasing age and the mean concentration of MIG in the older animals was significantly lower than in the younger age group. No significant correlations or mean differences in circulating levels of I-TAC, MDC, or RANTES were observed with age or between age groups, respectively. There also were no significant differences in means or correlation coefficients of these chemokine concentrations over age between males and females.

Concentrations of the growth factors, EGF, FGF-basic, G-CSF, IL2, and TGFα significantly correlated directly with age. Mean circulating levels of these growth factors also were statistically significantly higher in the older group than the younger group, with the exception that FGF-basic, although expressed at higher levels in the older group, exhibited a wide standard deviation to preclude reaching statistical significance. Conversely, TGFβ3 concentrations inversely correlated with age. There were no statistically significant correlation coefficients over age or significant differences in mean concentrations between the older and younger groups when evaluating GM-CSF, HGF, TGFβ1, TGFβ2, and VEGF. No gender differences were observed in correlation coefficients over age or mean concentrations between the older and young age groups for all of the growth factors assayed.

Among the anti-inflammatory cytokines, levels of IL1ra and IL4 significantly correlated directly with age, and mean concentrations were significantly higher in the older than younger group of animals. IL5 levels significantly correlated inversely with age and mean levels were significantly lower in the older group than the younger group of animals. Circulating levels of IL10 and IL13 did not correlate with age. There also were no differences between genders in correlation coefficients with age or mean levels of the anti-inflammatory cytokines.

To identify a group or panel of cytokines/chemokines expressed in levels that correlated to each other and in relation to aging, pairwise Pearson’s correlation analysis was performed among the circulating cytokines tested in common to both multiplex kits in the study (Table 5). A set of ten cytokines was identified that exhibited statistically significant correlation coefficients in each pairwise comparison within the group. These concordant cytokines included eight pro-inflammatory cytokines and chemokines, IFNγ, IL1β, IL6, IL12, IL15, TNFα, MCP1, and MIP1α, (inside boxed area), as well as two anti-inflammatory cytokines, IL1ra and IL4 (external boxed area).

**Table 5:** **Pairwise Pearson correlation between circulating cytokine and chemokine levels in rhesus macaques**^**a**^

	**IFN-γ**	**IL-1β**	**IL-6**	**IL-12/ 23 (p40)**	**IL-15**	**TNF-α**	**MCP-1**	**MIP-1α**	**IL-1ra**	**IL-4**	**MIP-1β**	**IL-17**	**MIF**	**Eotaxin**	**IL-8**	**I-TAC**	**MDC**	**MIG**	**RANTES**
**IFN-γ**																			
**IL-1β**	**0.406**																		
**IL-6**	**0.725**	**0.425**																	
**IL-12/23(p40)**	**0.408**	**0.763**	**0.341**																
**IL-15**	**0.476**	**0.853**	**0.434**	**0.768**															
**TNF-α**	**0.668**	**0.470**	**0.438**	**0.534**	**0.527**														
**MCP-1**	**0.276**	**0.637**	**0.332**	**0.466**	**0.561**	**0.286**													
**MIP-1α**	**0.689**	**0.788**	**0.664**	**0.712**	**0.779**	**0.582**	**0.507**												
**IL-1ra**	**0.377**	**0.855**	**0.376**	**0.893**	**0.843**	**0.509**	**0.597**	**0.772**											
**IL-4**	**0.320**	**0.932**	**0.395**	**0.814**	**0.828**	**0.441**	**0.626**	**0.771**	**0.927**										
**MIP-1β**	**0.723**	**0.449**	**0.565**	**0.374**	**0.445**	0.184	**0.262**	**0.690**	**0.355**	**0.351**									
**IL-17**	0.075	0.174	−0.003	**0.575**	**0.317**	0.155	0.132	0.306	0.475	**0.226**	0.166								
**MIF**	−0.086	0.263	−0.221	0.008	0.054	0.096	−0.063	−0.013	0.177	**0.456**	−0.092	0.015							
**Eotaxin**	0.344	**0.933**	0.113	−0.271	0.298	0.239	**0.672**	**0.366**	−0.170	−0.202	0.278	−0.155	0.163						
**IL-8**	−0.112	**−0.355**	−0.094	**−0.285**	**−0.317**	−0.085	−0.029	**−0.331**	**−0.308**	**−0.329**	**−0.303**	−0.115	**0.513**	−0.125					
**I-TAC**	**0.443**	0.222	**0.425**	−0.143	**0.518**	0.070	**0.397**	**0.473**	0.241	−0.233	0.356	0.148	0.094	**0.393**	−0.070				
**MDC**	−0.102	−0.160	−0.245	−0.034	−0.108	−0.125	−0.171	−0.171	0.086	0.142	−0.071	−0.126	−0.029	−0.211	0.024	−0.137			
**MIG**	0.039	0.191	−0.128	0.349	0.097	**0.376**	0.079	−0.006	−0.259	**−0.376**	0.023	0.138	−0.186	0.187	−0.263	0.121	0.023		
**RANTES**	−0.065	−0.162	−0.237	**0.730**	0.148	**0.415**	−0.085	0.137	**0.547**	0.271	0.004	**0.613**	0.225	−0.210	0.041	−0.181	−0.009	−0.020	

^a^ Pairwise Pearson correlation coefficients (r values) were measured among cytokines and chemokines using GraphPad Prism software. Statistically significant (*P* < 0.05) correlation coefficients are designated in bold text. The inner box designates concordant correlation coefficients that were significant for each pair of inflammatory cytokines and chemokines tested within the group and the outer box also includes the two anti-inflammatory cytokines with significant correlation coefficients between each pairwise evaluation.

**Figure 4:**
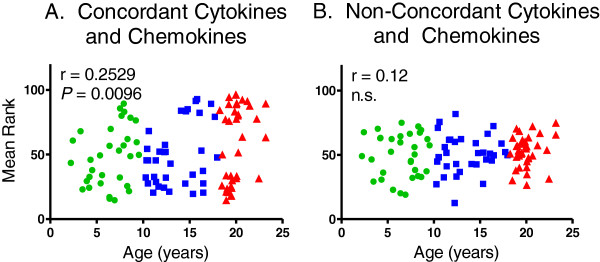
**Relationships between mean cytokine/chemokine rankings and age of rhesus macaques.** Animals were ranked from 1 (low) to 101 (high) for each positively correlating cytokine/chemokine (and in reverse for each inversely correlating factor). Mean rank values of the group of factors exhibiting concordant pairwise correlation coefficients (Panel **A**) and the remaining cytokines/chemokines (Panel **B**) were plotted against the age of each monkey. Pearson correlation coefficients were calculated between the mean rank values and age, and *P* < 0.05 was considered statistically significant.

Rhesus macaques, like humans, are outbred species and can express highly variable cytokine responses. As a result, incidentally high or low outlier concentrations of any single cytokine/chemokine in an individual animal may affect significance of correlation coefficients and comparison of means. This was also evident by the higher standard deviations in mean concentrations of several cytokines expressed in the older group of animals. To corroborate the pairwise correlation analysis and adjust for the possible effect of potential outlier cytokine/chemokine concentrations, animals were ranked from 1 (lowest concentration rank) to 101 (i.e. highest concentration rank based on the total number of animals tested in this study) for each cytokine expressing a positive correlation coefficient with age (and in reverse if expressing a negative correlation with age) and tied rankings were given the median rank for that range of animals. Mean ranks for the concordant pairwise cytokines associated with aging, as well as the non-concordant factors, were plotted in relation to age in Figure 4. A statistically significant correlation was observed between the mean rankings of the ten concordant cytokines identified in Table 5 over age (r = 0.2529; P = 0.0096) whereas no correlation was observed between mean ranks of the remaining non-concordant cytokines and age (r=0.12; n.s.). In addition, the mean rankings of the traditional inflammatory cytokines associated with aging, namely IFNγ, IL1β, IL6, IL12, and TNFα also exhibited a statistically significant correlation (r=0.1990; *P* = 0.0428; not shown) but this was less robust than the correlation expressed by the panel of ten pairwise concordant cytokine identified on Table 5.

## Discussion

For probably the first time in history, persons over the age of 65 outnumber children under 5 years of age [5]. With increasing life expectancy however, there has occurred an increase in infectious disease severity, neoplasia, and autoimmune disease as a consequence of immune senescence in the elderly [2,3]. This poses challenges to public health and medical care in the elderly for vaccination against infectious diseases and treatment of chronic diseases, respectively. For example, influenza and pneumonia are among the top ten causes of death in the elderly, yet influenza vaccination is only 17 – 53% effective in elderly adults compared to 70 – 90% efficacy in younger adults [11]. Defining mechanisms of immune senescence is expected to help generate intervention therapies and establish predictive markers to identify those individuals requiring supportive strategies such as vaccine boosting, for example.

Nonhuman primates provide a vital link for translating basic science research to applications in humans for improving well-being in the elderly [7,12]. Advantages to using nonhuman primates include genetic homology, physiology, behavior, and responses to infections and diseases that are shared with humans. In addition, nonhuman primates are outbred, and outdoor-housed animals accumulate environmental exposures similar to humans [9,13]. Clinical, medical, and pedigree information is available for captive nonhuman primates used in biomedical studies, as well. Medications and compliance of their use by elderly humans may affect interpretation of results from studies on natural biological aging, so studies using nonhuman primates may control for these effects. Nonhuman primates also serve as analogous models of humans with similar and often identical susceptibility to infectious agents. This provides another advantage because nonhuman primates can be experimentally challenged with infectious disease agents to validate vaccine or drug efficacy which usually cannot ethically be accomplished in humans. Further validating this nonhuman primate model in gerontology are reports that dietary restriction extending median and maximum lifespan in a wide range of animals such as worms, fruit flies, and mice, also extends life-span or delays onset of age-related chronic diseases in nonhuman primates, thus supporting translation to humans [14-17].

Chronic low-grade inflammation is associated with aging but questions remain whether inflammation is causal, coincidental, or consequential to aging, and which biomarkers of inflammation are relevant to studies on immune senescence [4,6,18,19]. Rhesus macaques are among the most commonly-used nonhuman primates in biomedical research and provide applicable models that simulate human physiology and inflammation related to aging [7,9,12,20]. A goal of this study therefore, was to correlate immunological parameters from blood samples of outdoor-housed rhesus macaques to develop a model of immune-senescence that can be used to study mechanisms relating inflammation and aging.

The rhesus macaques used in this study were housed outdoors and ranged from 2.5 to 24 years of age that is roughly equivalent to 8 – 77 years of age in humans. A cross-sectional experimental design was applied to correlate characteristics of blood, and especially cytokine/chemokine concentrations over age and compare means between older and younger animals to define significant characteristics associated with aging. Neonatal and infant animals were excluded so that developmental characteristics would not confound the correlations that often shift with maturation. In addition, exceptionally long-lived rhesus macaques aged 25 years or more (approximately equivalent to 80 years of age in humans) were not included because frail older animals will have died and characteristic trends of overall aging were reported to deviate or change in exceptionally longer-living older animals [21]. Excluding exceptionally longer-lived animals also helps distinguish correlates related to primary changes of aging from secondary changes affected by diseases of aging. The preponderance of females to males at an approximate ratio of 3:1 reflects the population demographics of the outdoor breeding colonies at the TNPRC and gender differences were only rarely observed in these studies.

Initial studies on blood chemistry and hematology performed on outdoor-housed animals were similar to results previously published on indoor-housed rhesus macaques. An extensive study and update by Smucny et al. describing characteristics of blood chemistry and hematology during aging in captive rhesus macaques identified significant declines in albumin, albumin/globulin, creatinine, MCV and MCH along with significant increases in alkaline/phosphatase, BUN, BUN/creatinine, Hct, Hgb, and RBC numbers [22,23]. In this current study, significant inverse correlations with age were reported for albumin, creatinine, and aspartate transaminase (AST). AST was not measured in the Smucny report and triglycerides were not measured in the study reported here. There also was no significant correlation between erythrocyte numbers and age in the outdoor-housed rhesus macaques of this study, and although mean Hct and Hgb levels were lower for females than males, there were no significant differences in correlation coefficients between genders over age.

No significant changes in circulating levels of WBC were reported in the Smucny studies over age whereas significant inverse correlations over age were observed for WBC in the studies reported here and as also reported for humans previously [22-24]. Results reported on the outdoor-housed macaques also demonstrated declines in circulating levels of lymphocytes, monocytes, neutrophils, eosinophils, and basophils over age. The decline in blood lymphocyte levels overall was reflected primarily from decreasing levels of B cells with age, rather than T cells or NK cells. Several reports suggested a consensus that overall T cell levels in blood do not change with age, as also observed here, and while variable results were reported in humans and mice regarding levels of NK and B cells, most of these studies described declining functional capabilities of these cells with aging [25-28].

Overall numbers of circulating CD4+ T cells and CD8+ T cells did not significantly correlate with age in this group of outdoor-housed macaques. Within the T cell populations, however, there was a significant direct correlation between CD8+ effector memory T cells and significant inverse correlations between CD4+ and CD8+ naïve T cell populations with age. These findings are similar to reports by others [29-33], including a study on indoor-housed rhesus macaques reported by Cicin-Sain and colleagues comparing mean levels between younger (aged 6–9 years old) and older (aged 18 – 24 years old) rhesus macaques. In addition, we observed a significant correlation between increasing levels of DP (CD4+CD8+) T cells in blood with increasing age, and numbers of DP effector memory cells statistically significantly correlated with age while numbers of DP naïve and central memory cells did not significantly correlate with age.

Identifying biomarkers of aging may be important for predicting vaccine failure or susceptibility to infectious disease pathogens, cancer or other diseases that are especially problematic in the elderly. Chronic low-grade inflammation is considered a hallmark of aging, and multiplex cytokine analysis was applied in attempt to identify a panel or group of systemic cytokines that together significantly correlate with aging. Such a panel of cytokines is expected to be more consistent than an individual cytokine/chemokine level for selecting individuals with relatively higher and lower levels of inflamm-aging into research studies. The ability to select groups of animals with high and low inflammation status will be important to test hypotheses and mechanisms relating cause and effect between inflammation status and aging. This would also open the door to developing strategies to restore immune competence or delay onset of immune senescence.

This study is among the first to apply multiplex cytokine analyses in rhesus macaques for developing a model of immune senescence. The outdoor cohort of macaques exhibited increasing levels of several pro-inflammatory cytokines with age, including IFNγ, IL1β, IL6, IL12, IL15, MIF, sCD40L, and TNFα with age, similar to observations by others [18,34-36]. Of interest was the observation that while circulating levels of IFNγ, IL6, IL15, MIF, and CD40L correlated directly with age, *t* test comparisons between mean levels in the older group of macaques (aged 18 – 24 years) were not significantly higher than those in the younger group of macaques (aged 2 – 9 years), in part due to higher variability exhibited by wider standard deviation values. Such findings may be especially prominent in studies of outbred populations such as humans and nonhuman primates than perhaps would be expected to occur inbred strains of mice. The higher variation in older groups might also explain why others reported no significant differences between younger humans (less than 45 years of age) and older humans (65 years or more) when comparing mean circulating levels of cytokines such as IFNγ, IL6, IL12, or TNFα after multiplex testing and applying *t* test analyses [37].

Individual cytokines behave in complex, redundant, and pleiotropic mechanisms making it difficult to relate any single pro-inflammatory cytokine to biological aging. In addition, the increasing standard deviations in mean concentrations of several circulating pro-inflammatory cytokines suggested that not all animals exhibited systemic inflamm-aging at the same rate as chronological aging. A goal of this study was to establish a basis for selecting animals exhibiting relatively higher and lower levels of cytokines/chemokines associated with inflamm-aging so that subsequent studies can be planned to test how inflammation affects susceptibility and development of chronic diseases in the elderly. In attempt to define a group of cytokines/chemokines that reflect immune senescence, Pearson pairwise correlation analysis was applied to each of the cytokines assayed in the multiplex testing platform. A “concordant” set of cytokines/chemokines was identified whose levels correlated with aging and that also exhibited significant pairwise correlations with each of the other factors of this group. These included the pro-inflammatory cytokines IFNγ, IL1β, IL6, IL12, IL15, MCP1, MIPα, and TNFα. Of interest was the observation that two anti-inflammatory cytokines, IL4 and IL1ra, also exhibited significant correlations with each of the other concordant cytokines in this group. The increased levels of circulating IL4 and IL1ra levels correlating with age may indicate repair responses to inflammation or may reflect dysregulation of cytokines and chemokines purported to occur during immune-senescence [36,38]. To ascertain that there were no outlier cytokine concentrations that could confound interpretation of these results, mean rankings rather than cytokine concentrations were analyzed and found to significantly correlate directly with age whereas mean rankings of the remaining cytokines as a group did not correlate with aging. Furthermore, the panel of 10 concordant cytokines exhibited a more robust correlation with age than did a smaller group of number of cytokines, IFNγ, IL1β, IL6, IL12, and TNFα, that are routinely associated with inflamm-aging. The use of mean ranks compressed the dynamic range of analyses to generate more conservative correlation coefficients, but demonstrated that the mean rankings of cytokines containing both pro- and anti-inflammatory activities exhibited greater statistical significance than the mean rankings of the five pro-inflammatory cytokines commonly associated with aging. These results provide a model to compare animals exhibiting relatively higher versus lower levels of these cytokines and chemokines to relate chronological aging with biological aging as well as address impact on susceptibility to chronic diseases typically associated with aging. Also of interest was the observation that a few of the younger animals exhibited relatively higher inflammatory cytokine rankings, and longitudinal studies could be performed to determine if these higher cytokine/chemokine-ranking younger animals were responding to recent infections or are pre-disposed to biologically age faster than their lower ranking cohorts.

Risk factors associated with immune senescence have included chronic infections with cytomegalovirus (CMV) and obesity. We compared body weights of the older 18 – 24 year-olds with high and low ranking circulating chemokine/cytokine levels (above or below 50, respectively; Figure 4, panel A) among females (8.99 kg ± 0.46 vs 8.02 kg ± 0.29, respectively) and males (14.35 kg ± 1.46 vs 12.35 ± 1.71, respectively) and found no statistically significant differences within each gender. The rhesus macaques used in this study are housed outdoors and are thus less sedentary than indoor-housed animals which could explain this observation. In addition, animals over age 24 were not used in this study to preclude changes that may be associated with chronic diseases of aging rather than aging itself. Chronic CMV infection is believed to promote the higher levels of effector-memory T cells that affect the overall immunological cell population profiles that shift with aging. Based on a summary of the second CMV and immunosenescence workshop, many questions remain about the impact of CMV infection on the aging immune system and whether treatment to clear CMV, for example, would delay or reverse immune senescence [39]. Monkeys are not routinely tested serologically for CMV at the Tulane National Primate Research Center, but a limited study of the SPF colony at the Tulane National Primate Research Center several years ago determined that approximately 20% of the animals were CMV seronegative and that these were all among the younger group of rhesus macaques. All of the animals in the older group were thus highly likely to be CMV seropositive suggesting that CMV infection alone was not the cause for the varied inflammation status observed in the older rhesus macaques. Further studies, however, are needed to corroborate this.

The results of this study confirm and extend the benefits afforded by using nonhuman primates to study aspects of aging related to systemic pro- and anti-inflammatory cytokines/chemokines. From the literature, there seems to be consensus that increased levels of circulating inflammatory cytokines/chemokines are indicators of increased biological aging and risk for frailty in the elderly [12,18,38,39]. It is unclear, however, whether age-correlating changes in these cytokine/chemokine levels represent biomarkers of aging, contribute to chronic diseases of aging, are produced as a consequence of developing diseases of aging, or combination of these possibilities. Continued studies using this nonhuman primate model will allow us to test whether the higher ranking animals putatively exhibiting “inflamm-aging” are more susceptible than the relatively lower ranking animals to experimental infectious disease or vaccine failure. Such a model can then be used to discern between primary changes related to aging from secondary effects of diseases of aging that would also affect inflammation.

## Conclusions

The results of this study extend the findings by others demonstrating significant correlations between declining naïve T cells, increasing effector memory CD8+ T cells, and increasing levels of pro-inflammatory cytokines/chemokines in blood and age in outdoor-housed rhesus macaques. New information includes the application of multiplex quantification to identify a panel of chemokines/cytokines that collectively correlate with aging. Assessing cytokine/chemokine rankings instead of circulating concentrations further corroborated the correlation between this panel of factors with aging that will facilitate selection of animals exhibiting relatively higher and lower inflammation status. Such a model will thus help define predictors of aging and address mechanisms relating inflammation to aging.

## Competing interests

The authors declare no financial or non-financial competing interests in the work presented in this study.

## Authors’ contributions

ESD participated in planning the research, the multiplex cytokine studies, statistical analyses, and preparation of the manuscript. CS participated in planning the research, performed the flow cytometry experiments, contributed to analyses of flow cytometry data, and assisted with preparation of the manuscript. LCB participated in the research planning, clinical data analyses, multiplex cytokine assays, and preparation of the manuscript. IAK participated in planning the research, interpretation of results, and preparation of the manuscript. MJK participated in planning the research, evaluation of flow cytometry data, and preparation of the manuscript. All authors read and approved the final manuscript.
